# Appropriate Fat Supplementation in High-Starch Diets Involved in the Modification of Fatty Acids Profile, Amino Acids Composition, and Antioxidant Capacity of Adult Nile Tilapia (*Oreochromis niloticus*) Muscle

**DOI:** 10.1155/anu/7139771

**Published:** 2025-03-20

**Authors:** Jianmin Zhang, Ningning Xie, Ming Jiang, Lixue Dong, Hua Wen, Juan Tian

**Affiliations:** Key Laboratory of Freshwater Biodiversity Conservation, The Ministry of Agriculture and Rural Affairs, Chinese Academy of Fishery Sciences Yangtze River Fisheries Research Institute, Wuhan, China

**Keywords:** antioxidant capacity, fat level, flavor, high starch diet, nutritional value

## Abstract

Tilapia industry has faced great challenges due to the replacement of high-quality protein sources by a high proportion of starch. Meanwhile, the level of dietary fat is gradually reduced with the increase of oil price. High starch diets have been proved to have negative effects on flesh quality in previous studies, but the effects of fat remain unclear. The objective of the present study was to ascertain whether fat level is a requisite factor in the flesh quality of adult fish under conditions of high-starch diet feeding. The study involved adult Nile tilapia (*Oreochromis niloticus*) with an initial body weight (IBW) of 168.58 ± 2.01 g, which were fed a standard (CON) diet, a high-starch-low-fat (HSLF) diet, and a high-starch-moderate-fat (HSMF) diet for 10 weeks. The results demonstrated that the high starch diets significantly decreased the hardness, chewiness, springiness, and gumminess of muscle. HSLF diet led to a significant reduction in the weight gain rate (WGR), accompanied by an increase in crude fat content and a decrease in glycogen content in the muscle. The HSLF diet resulted in a reduction in the levels of polyunsaturated fatty acids (PUFAs), essential amino acids (EAAs), and flavor amino acids (FAAs) in the muscle tissue. Furthermore, it influenced muscle texture by reducing collagen content, fiber density, and sarcomere length. The muscle antioxidant capacity was diminished by affecting the total antioxidant capacity (T-AOC), catalase (CAT) activity, and superoxide dismutase (SOD) activity, as well as the expression levels of related genes (*SOD*, *CAT*, and nuclear factor erythroid 2 like 2 (*nrf2*)). In contrast, the HSMF diet did not have a detrimental impact on growth performance, yet it did result in a significant increase in glycogen content, hydroxyproline (Hyp), PUFAs, EAA, and FAA in muscle tissue. Moreover, the HSMF diet was observed to markedly elevate the antioxidant capacity of the muscle. It can be concluded that high-starch diet can significantly affect flesh quality by affecting the texture and muscle nutrients, as well as decreasing antioxidant capacity. Nevertheless, the inclusion of an adequate quantity of fat may prove an effective means of counteracting these unfavorable outcomes.

## 1. Introduction

Aquaculture, as the fastest growing sector in global food production, has been improving the nutritional levels of farmed species to meet the demand for high quality seafood in recent years [1]. Nile tilapia (*Oreochromis niloticus*) is one of the most widely farmed species in the world which has partially solved the problem of food shortage in developing countries [2, 3]. However, with the rapid development of aquaculture industry and the depletion of fishery resources, there is an increasing strain on high-quality feed ingredients [4]. The current situation faced by tilapia industry is to minimize the inclusion of protein and lipid sources in feed formulations to reduce costs. Carbohydrates are economic energy source that could spare protein for growth, and therefore, have been progressively adopted [5]. Starch are typical energy-providing carbohydrate elements. The glycosidic bond of amylose and amylopectin can be hydrolyzed by amylase to produce glucose for use [6]. Corn starch and wheat flour are frequently-used materials that are added directly or supplemented after gelatinization [7–9]. In tilapia, the representative species of omnivorous fish, dietary starch levels have surged to 30% and continued to rise in recent years [10–12]. High starch diets have achieved remarkable results in saving formulation cost. However, the persistent intake poses threat to the growth and health [13].

As energy storage tissue and edible part, the texture of fish muscle is closely associated with the growth state and fish nutritional status and is also greatly affected by dietary starch levels [14–16]. Studies on blunt snout bream (*Megalobrama amblycephala*) and grass carp (*Ctenopharyngodon idellus*) have revealed that high starch diet had a significant impact on amino acid composition and fatty acid composition [17]. A high starch diet may cause lower protein efficiency rate and lead to abnormal lipid accumulation that eventually influence the deposition of nutrients in muscle tissue [18].

Although the evidence that excessive starch intake may contribute to the increase of fat synthesis, the essential fatty acids can only be obtained through external supplementation [19]. However, dietary fat has been continued to decrease in recent years to saving costs, which influenced the sensory quality of muscle [20, 21]. Low fat diets also reduce the contents of essential omega-3 polyunsaturated fatty acids (*n*−3 PUFAs) and the absorption of fat-soluble vitamins which affect the growth of muscle tissue [19, 22]. Besides, the absence of fat can reduce antioxidant substances and increase the risk of muscle oxidation [23]. Therefore, the appropriate dietary fat level is crucial for ensuring the flesh quality. Previous studies have indicated that moderate fat level (76.6–87.9 g/kg) could alleviate the liver and intestinal damage of tilapia caused by high starch diet [24–26]. However, whether moderate fat levels could improve the flesh quality under high starch condition and the underlying mechanisms remain unclear. In the present study, tilapia were fed standard (CON) diet, high-starch-low-fat (HSLF) diet, and high-starch-moderate-fat (HSMF) diet, respectively, to explore the diet effects on flesh quality. The aim of the present study was to provide valuable insights for enhancing the muscle quality of cultured adult Nile tilapia and improve the scientific feed formulation to reduce costs and increase efficiency.

## 2. Materials and Methods

### 2.1. Animal Ethics

Fish management and sampling protocols were authorized by the Animal Experimental Ethical Inspection of Laboratory Animal Centre, Yangtze River Fisheries Research Institute, Chinese Academy of Fishery Sciences. The protocol number is YFI20200801.

### 2.2. Experimental Diets

The optimal starch and fat requirements for tilapia are 300–360 g/kg and 70–100 g/kg, respectively [27]. In the present study we used a CON diet containing 350 g/kg corn starch and 80 g/kg oil. Two high starch diets were prepared, including the HSLF diet (550 g/kg corn starch and 30 g/kg oil) and the HSMF diet (550 g/kg corn starch and 80 g/kg oil). Diet formulation of each group is presented in Table 1. Ingredients were manufactured into feeds with the length of 3 mm and the diameter of 2 mm after grinding, sieving, mixing, pelleting, and drying. The prepared pellet feeds were stored at −20°C for further use. The method for preparing the diet has been fully explained in our previous published research paper [28].

### 2.3. Experimental Fish and Procedure

Experimental adult tilapia were purchased from National Tilapia Fish Hatchery (Guangxi province, China) and temporarily cultured with CON diet for 2 weeks after disinfection. The feeding trial was conducted in recirculating aquaculture system of Yangtze River Fisheries Research Institute. There were three experimental groups with three replicates each group to feed the CON diet, the HSMF diet, and the HSLF diet. After 24 h starvation, 90 experimental adult tilapia from the same batch were randomly distributed to nine tanks (300 L water volume) with 10 fish per tank. During the experimental period, the water temperature was 25–28°C, the water pH was 8.1–8.3, the dissolved oxygen was greater than 5 mg/L, and the ammonia nitrogen was less than 0.05 mg/L. Experimental fish were hand-fed to satiation three times daily at 8:30 to 9:00, 12:30 to 13:00 and 17:00 to 17:30. The water volume was renewed about 30% daily and residual feeds were collected to calculate feed conversion ratio (FCR) and removed to maintain water quality.

### 2.4. Sampling

The initial body weight (IBW) were measured before the feeding trial. At the end of the feeding trial, all experimental fish were starved for 24 h and the final body weight (FBW), weight gain rate (WGR), and condition factor (CF) were measured and calculated. After anesthetization, the axial muscle above the lateral line of six adult tilapia per tank were dissected with different sizes. A total of 18 muscle samples per tank were collected. Six 1.0 cm × 1.0 cm × 0.5 cm muscle samples were used for the detection of texture. Six 0.5 cm × 0.5 cm × 0.5 cm muscle samples were preserved in 4% polyformaldehyde for paraffin section and six 0.2 cm × 0.2 cm × 0.2 cm muscle samples were preserved in 2.5% glutaraldehyde for ultrathin section. The muscle of remaining adult tilapia per tank were dissected and immediately frozen into liquid nitrogen for the determination of contents of amino acids and fatty acids, enzyme activities, and gene expression. Liver, mesenteric fat, and visceral mass were separated and weighed to calculate hepatosomatic index (HSI) and mesenteric fat index (MFI), and viscerosomatic index (VSI).

### 2.5. Analytical Methods

#### 2.5.1. Proximate Composition Analysis

Moisture of diets was determined by the loss on drying method (GB/T 5009.3-2016). Moisture of muscle was determined using vacuum freeze dryer (CHRIST, Germany). Crude protein, crude fat, and ash were determined by the Kjeldahl method (GB/T 5009.5-2016), the Soxhlet method (GB/T 5009.6-2016), and the muffle furnace method (GB/T 5009.4-2016), respectively. Dietary carbohydrate level was determined by spectrophotometric method (DB12/T 847-2018). Dietary gross energy was determined using an oxygen bomb calorimeter (SDC311, Hunan Sundy Science and Technology Co., Ltd., Changsha, Hunan province, China).

#### 2.5.2. Muscle Texture Detection

Muscle hardness, chewiness, springiness, gumminess, and resilience were evaluated using texture profile analysis method by TVT-300XP texture analyzer (Perten Instruments, Beijing, China). Hardness is indicator of taste which directly affects chewiness, springiness, and gumminess in texture profile analysis. Chewiness was defined as adhesiveness plus springiness that can be interpreted as the energy required to chew solid food. Springiness is the height at which food can be recovered between the end of the first bite and the beginning of the second. Gumminess is defined as hardness plus cohesiveness which can describe the taste of semisolid foods. Resilience is defined as the ratio of the area before the deformation target to the area after the deformation target during the first pressing down. The detection was performed with a cylindrical probe P-cy5s with pretest speed of 2 mm/s, post-test speed of 2 mm/s, test speed of 1 mm/s, interval time of 5 s, compression ratio of 60%, and data acquisition rate of 200 pps. The water holding capacity is the ability of proteins to absorb and retain water in protein tissues, which was detected according to previous publications [29].

#### 2.5.3. Muscle Hydroxyproline (Hyp) Content Determination

The Hyp content of three muscle samples per tank were determined according to published paper [30]. Briefly, the 1 g muscle was accurately weighed and homogenized with 9 mL precooled distilled water. Then the precooled NaOH (0.2 M) was added into the homogenate and oscillated at 4°C for 4 h, followed by centrifugation under 10000 × *g* for 30 min at 4°C. The supernatant was separated to obtain alkaline-soluble Hyp. The sediment (alkaline-insoluble Hyp) was transferred into 3 mL hydrochloric acid (6 M) in ampoule and hydrolyzed at 110°C for 20 h.

#### 2.5.4. Antioxidant Capacity Evaluation

The supernatant used for testing were obtained by homogenization (0.5 g muscle tissue and 4.5 mL deionized water) and centrifugation (3000 × *g*, 10 min, 4°C). The follow indices were measured with commercial kits (Nanjing Jiancheng Bio-Engineering Institute, Nangjing, China).

The total protein (TP) content was determined by the TP quantitative assay with coomassie brilliant blue method and calculated by measuring theabsorbance of solution at 595 nm.

Total antioxidant capacity (T-AOC) was quantified measured using Trolox as standard and T-AOC can be expressed in terms of the amount of Trolox.

Catalase (CAT) activity was determined using the ammonium molybbate method. The decomposition of H_2_O_2_ by CAT can be quickly stopped by the addition of ammonium molybbate and the remaining H_2_O_2_ reacted with ammonium molybbate to form a pale yellow complex. The content of the complex was determined at 405 nm.

Superoxide dismutase (SOD) activity was assayed with hydroxylamine method. Superoxide anion free radicals produced by the reaction system of xanthine and xanthine oxidase oxidize hydroxylamine to form nitrite and demonstrate purple and red. The absorbance values were measured at 550 nm.

#### 2.5.5. Histological Analysis

**Figure 1:**
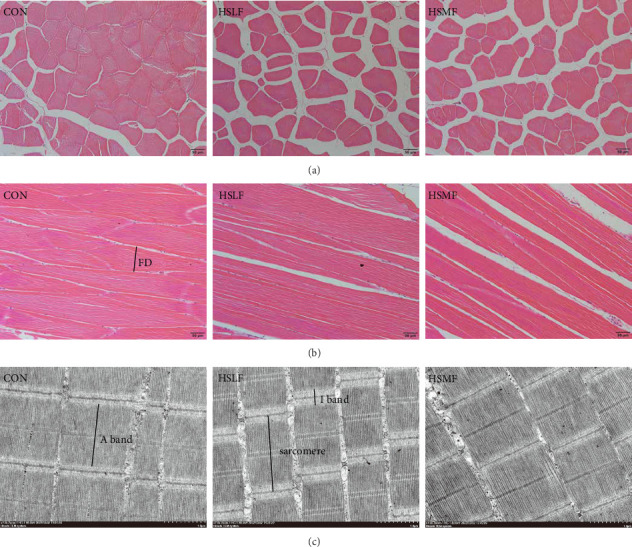
Muscle tissue sections of Nile tilapia under optical microscope and electron microscope. (A) Horizontal section, (B) longitudinal section, and (C) electron microscopy of muscle fibers. FD: fiber diameter.

The paraffin section and hematoxylin and eosin staining were carried out according to the methods of previous study using the 0.5 cm × 0.5 cm × 0.5 cm muscle samples [31]. The stained sections were observed with optical microscope (Olympus BX53, Tokyo, Japan). Ultrathin sections prepared with 0.2 cm × 0.2 cm × 0.2 cm muscle samples were prepared and observed with transmission electron microscope (HT7800, Tokyo, Japan) in Wuhan Servicebio Technology Co., Ltd. The fiber diameters, fiber density, sarcomere length, I band length, and A band length were measured with Image-J software. Fiber density was calculated according to the number of muscle fibers of unit area. Fiber diameters, sarcomere length, I band length, and A band length were measured, as shown in Figure 1.

#### 2.5.6. Fatty Acid Composition Determination

The 0.4 g freeze-dried muscle was weighed, vortexed, and mixed with 4 mL isooctane for 30 s, and shaken at 25°C overnight for extraction. The extracted solution was mixed with 8 mL 2% sodium hydroxide solution in methanol and slightly boiled for 40 min, mixed with 7 mL 15% trifluoro (methanol) boron and slightly boiled for 20 min, and mixed with 20 mL n-heptane and slightly boiled for 1 min, successively. Saturated sodium chloride solution was then added and stood to layering. A 5 mL of the upper n-heptane extraction solution was separated and mixed with 5 g anhydrous sodium sulfate. The mixture was shaken for 1 min and stood for 5 min. The supernatant was then extracted into loading bottle. Fatty acid contents were determined with gas chromatograph (Agilent 7890 A, Beijing, China). Polydicyanpropyl siloxane capillary column with strong polar stationary phase was used with 1 μL of injection volume and 270°C and 280°C with the injection and detection port temperatures. The temperature was set up as follows: first 13 min at 100°C, and then the temperature was increased to 180°C at a rate of 10°C per min and maintained for 6 min. Then, the temperature was increased to 200°C at a rate of 1°C per min and stayed there for 20 min. After reaching 230°C at a rate of 4°C per min, the temperature was maintained for 10.5 min. The carrier was nitrogen gas, and the split ratio was 100:1. The fatty acid content was calculated according to the ratio of the peak area of different fatty acids to the peak area of the internal standard (C11:0) [32].

#### 2.5.7. Amino Acid Composition Determination

Bound amino acid contents were determined as follows method: The 0.1 g freeze-dried muscle was accurately weighed and transferred into 12 mL hydrochloric acid (6 M) in ampoule and hydrolyzed at 110°C for 24 h. After cooling, the hydrolysate was filled to 100 mL with ultrapure water, and 2 mL was placed in a vial and dried in a vacuum dryer at 60°C for 24 h. After complete evaporation, 2 mL distilled water was added and returned to the vacuum dryer for 24 h. The precipitate was dissolved in 8 mL hydrochloric acid (0.1 M), filtered through a 0.22 μm aqueous phase filter membrane into amino acid loading bottle, and detected with an amino acid analyzer (HITICHI L-8900, Tokyo, Japan) [32].

Free amino acids were determined as follows method: The 0.1 g muscle was accurately weighed and added into 3 mL sulfosalicylic acid (10%) for homogenization. The 400 μL serum was added into 1.2 mL sulfosalicylic acid (10%) for homogenization and centrifugation (13000 × *g*, 15 min, 4°C). The supernatant was extracted, filtered through a 0.22 μm aqueous phase filter membrane into amino acid loading bottle and detected with an amino acid analyzer (HITICHI L-8900, Tokyo, Japan) [32].

#### 2.5.8. cDNA Synthesis and RT-qPCR

Muscle samples were immediately transferred from −80°C to liquid nitrogen for grinding. The procedure of total RNA extraction was conducted using TRIzol (Life Technologies, Carlsbad, CA, USA) under the guidance of instruction book. After integrity and concentration testing, RNA was reverse transcribed to cDNA using PrimeScript RT kit (Takara, Dalian, Liaoning province, China) under the guidance of instruction book. In this study, qPCR experiments were performed using at least three biological replicates. The RT-qPCR was carried out using the SYBR Premix Ex Taq kit (Takara, Dalian, Liaoning province, China). The 20 μL reaction system was prepared with 10 μL SYBR Premix Ex Taq, 0.8 μL forward primer (10 μM), 0.8 μL reverse primer (10 μM), 0.4 μL 50 × ROX Reference Dye Ⅱ, 6 μL DEPC water, and 2 μL cDNA template. The RT-qPCR program was set up as follow: 95°C for 5 min, followed by 40 cycles of 95°C for 30 s, 58°C for 30 s, and 72°C for 30 s. Then 72°C for 5 min. A standard curve was constructed using six tenfold gradient dilutions of cDNA. After the qPCR reaction, the log value of the serial dilution of the template was used as the *X*-axis, and the corresponding Ct value was used as the *Y*-axis to plot the standard curve. Primer sequences used in the present study was designed and synthesized by Sangon Biotech (Shanghai, China) according to the to the corresponding sequences in GenBank. The mRNA expression levels of myogenic differentiation 1 (*myod*), located on the complementary strand between positions 3,598,005 and 3,600,314, myogenin (*myog*), located between positions 25,640,809 and 25,643,049, myogenic factor 5 (*myf5*), located between positions 15,815,227 and 15,818,324, and myogenic factor 6 (*myf6*), located between positions 15,824,384 and 15,838,147, were determined to assess the development of myofibers. Additionally, the mRNA expressions of nuclear factor erythroid 2 like 2 (*nrf2*), located between positions 17,007,276 and 17,013,930, CAT (*cat*), located between positions 45,270,104 and 45,279,554, and SOD 1 (*sod*), were detected to evaluate the antioxidant capacity of muscle (Table 2). The 2^−*ΔΔ*Ct^ method was applied to normalize fluorescence data to *β-actin* [33]. Briefly, The Ct values of target genes were normalized to those of *β-actin*, and relative expression levels were calculated using the *ΔΔ*Ct method. Specifically, *Δ*Ct was calculated as Ct (target) − Ct (*β-actin*), and *ΔΔ*Ct was determined as *Δ*Ct (experimental) − *Δ*Ct (control), with relative expression quantified as 2^−*ΔΔ*CT^.

### 2.6. Calculations and Statistical Analysis

  
$$\begin{array}{c} \text{WGR}\left(\%\right)=\left(\text{FBW}-\text{IBW}\right)/\text{ IBW}\times 100\%. \end{array}$$

  
$$\begin{array}{c} \text{FCR}=\text{ feed intake}/\left(\text{FBW}-\text{IBW}\right). \end{array}$$

  
$$\begin{array}{c} \text{HSI}\left(\%\right)=\text{ hepatic weight}/\text{FBW}\times 100\%. \end{array}$$

  
$$\begin{array}{c} \text{MFI}\left(\%\right)=\text{ mesenteric fat weight}/\text{FBW}\times 100\%. \end{array}$$

  
$$\begin{array}{c} \text{VSI}\left(\%\right)=\text{ visceral wet weight}/\text{final body weight}\times 100\%. \end{array}$$

  
$$\begin{array}{c} \text{CF}\left(\text{g}/{\text{cm}}^{3}\right)=\text{ FBW}/{\left(\text{FBL}\right)}^{3}. \end{array}$$

Statistical analysis was carried out with IBM SPSS Statistics 23.0 (SPSS Inc., USA). Data were subjected to one-way analysis of variance (ANOVA) followed by Tukey's test. *p* < 0.05 was applied to show statistically significant differences. Results were presented as mean ± standard deviation (SD).

## 3. Results

### 3.1. Growth Performance

Growth performance of tilapia is shown in Table 3. The HSLF diet resulted in a significant decrease in FBW and WGR and significant increase in FCR, MFI, and VSI (*p* < 0.05). HSMF diet did not have a negative effect on WGR and FCR compared to the CON group (*p* > 0.05), but also induced significant enhancement of HSI, MFI, and VSI (*p* < 0.05). The CF was significantly higher when fish were fed the HSMF diet (*p* < 0.05).

### 3.2. Muscle Proximate Composition

As shown in Table 4, neither HSLF nor HSMF diets had significant effects on the moisture, crude protein, or ash (*p* > 0.05). Compared with the CON group, HSLF diet significantly increased crude fat and decreased the glycogen content, while HSMF diet significantly increased the glycogen content (*p* < 0.05).

### 3.3. Muscle Texture

Results of indices related to muscle texture are presented in Table 5. Both HSLF and HSMF diets significantly decreased the hardness, chewiness, springiness, and gumminess of muscle compared to the CON group (*p* < 0.05). Only HSLF diet remarkably decreased the resilience of muscle (*p* < 0.05).

### 3.4. Muscle Hyp Content

**Figure 2:**
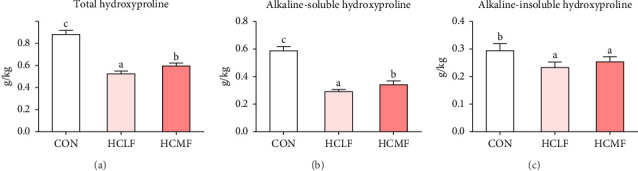
The contents of total hyroxyproline (A), alkaline-soluble hyroxyproline (B), and alkaline-insoluble hyroxyproline (C) of Nile tilapia muscle tissue. The hyroxyproline values are means (*n* = 3), with their standard deviations (SDs) represented by vertical bars. Bar graphs of the same color, but with different letters are significantly different (*p* < 0.05).

Hyp contents are exhibited in Figure 2. Compared with the CON group, both HSLF and HSMF diets significantly decreased the contents of total Hyp, alkaline-soluble Hyp, and alkaline-insoluble Hyp (*p* < 0.05). Compared with the HSLF group, the contents of total Hyp and alkaline-soluble Hyp were remarkably higher in HSMF group (*p* < 0.05).

### 3.5. Muscle Histology and Related Gene Expression

**Figure 3:**
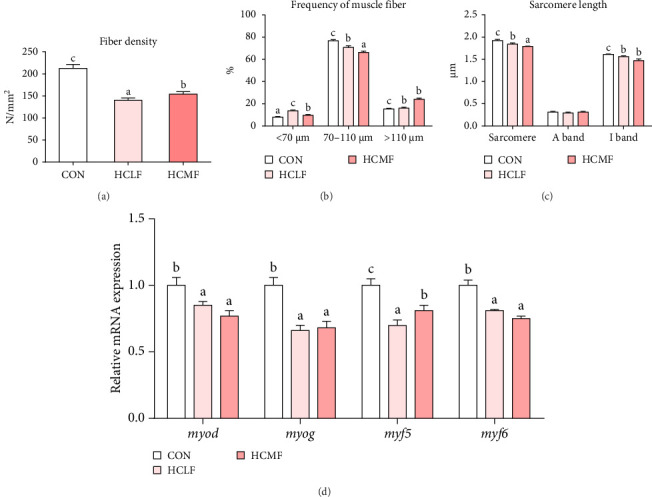
Muscle fiber characteristics indexes and mRNA expression levels about muscle fiber development of Nile tilapia. (A) Fiber density, (B) frequency of muscle fiber, (C) sarcomere length, and (D) mRNA expression levels about muscle fiber development of Nile tilapia. Data values are means (*n* = 3), with their standard deviations (SDs) represented by vertical bars. Bar graphs of the same color, but with different letters are significantly different (*p* < 0.05). *myf5*, myogenic factor 5; *myf6*, myogenic factor 6; *myod*, myogenic differentiation 1; *myog*, myogenin.

Both HSLF and HSMF diets significantly influenced the histology and development of muscle tissue of tilapia (Figure 1). Compared with the CON group, HSLF and HSMF diets significantly reduced the fiber density of muscle (*p* < 0.05; Figure 3A). The proportion of fiber with diameters ranged from 70–100 μm was significantly decreased while the proportion of fiber with diameters less than 70 μm was remarkably increased (*p* < 0.05; Figure 3B). The sarcomere length and A band length were significantly lower (*p* < 0.05; Figure 3C). Furthermore, HSLF and HSMF diets significantly downregulated the mRNA expression levels of *myod*, *myog*, *myf5*, and *myf6* (*p* < 0.05). The level of *myf5* was higher in HSMF group compared to HSLF group (*p* < 0.05; Figure 3D).

### 3.6. Muscle Fatty Acids Composition

A total of 24 fatty acids were detected in the present study, including eight saturated fatty acids (SFAs), seven monounsaturated fatty acids (MUFAs), and nine PUFAs (Table 6). Compared with the CON group, HSLF diet remarkably increased the contents of SFAs and MUFAs, but decreased the content of PUFAs, especially *n*−3 PUFAs represented by C20 : 5*n*−3 (EPA) and C22 : 6*n*−3 (DHA; *p* < 0.05). HSMF diet also significantly decreased the contents of PUFAs, however, compared with the HSLF group, the contents of *n*−3 PUFAs and DHA were remarkably higher (*p* < 0.05). Moreover, the ratio of *n*−3 PUFAs to *n*−6 PUFAs and PUFAs to SFAs were remarkably lower in HSLF group compared to other groups (*p* < 0.05).

### 3.7. Muscle Bound Amino Acids Composition

A total of 17 bound amino acids were detected (Table S1). There were no significant differences between three groups on level of individual amino acid. However, compared with the CON group, HSLF diet significantly decreased essential amino acids (EAAs) levels, while the level of non-EAAs (NEAAs) was enhanced (*p* < 0.05; Figure S1). HSMF also increased NEAA level, but had no significant impact on EAA level (Figure S1).

### 3.8. Muscle Free Amino Acids Composition

**Figure 4:**
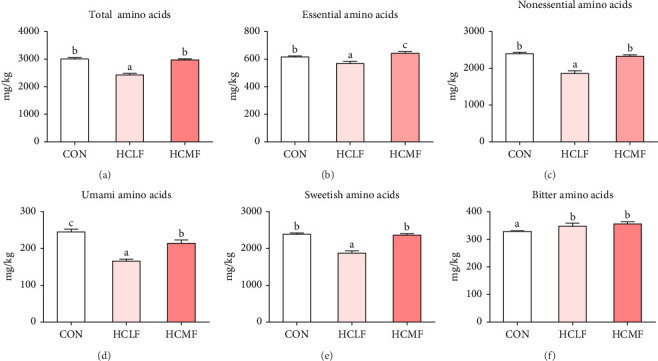
Muscle free amino acid levels of Nile tilapia. (A) Total amino acids (TAAs), (B) essential amino acids (EAAs), (C) non-EAAs (NEAAs), (D) umami amino acids (glutamate and aspartate), (E) sweetish amino acids (lysine, threonine, alanine, glycine, proline, and serine), and (F) bitter amino acids (arginine, histidine, isoleucine, leucine, methionine, and valine). Data values are means (*n* = 3), with their standard deviations (SDs) represented by vertical bars. Bar graphs of the same color, but with different letters are significantly different (*p* < 0.05).

A total of 16 free amino acids were detected (Table 7). Compared with the CON group, HSLF diet significantly decreased levels of total amino acids (TAA), EAA, and NEAA (*p* < 0.05; Figure 4). Isoleucine, lysine, methionine, threonine, threonine, alanine, glutamate, proline, and tyrosine levels were significantly lower, while arginine, histidine, glycine, and serine levels were significantly enhanced (*p* < 0.05; Table 7). HSMF diet significantly increased the level of NEAA (*p* < 0.05). Isoleucine, threonine, glutamate, serine, and tyrosine levels were significantly lower, while arginine, histidine, leucine, lysine, alanine, aspartate, and glycine levels were significantly higher (*p* < 0.05). Compared with the HSLF group, the levels of TAAs and EAAs were remarkably higher when fish fed with HSMF diet (*p* < 0.05; Figure 4). Furthermore, high starch diets influenced the muscle flavor amino acids (FAAs). Compared with the CON group, HSLF diet significantly decreased the levels of umami amino acids and sweetish amino acids while increased the level of bitter amino acids (*p* < 0.05; Figure 4). HSMF diet had same effects on the levels of umami and bitter amino acids as HSLF diet (*p* < 0.05; Figure 4). However, the sweetish amino acids returned to CON levels in HSMF group (*p* < 0.05; Figure 4).

### 3.9. Muscle Antioxidant Capacity

**Figure 5:**
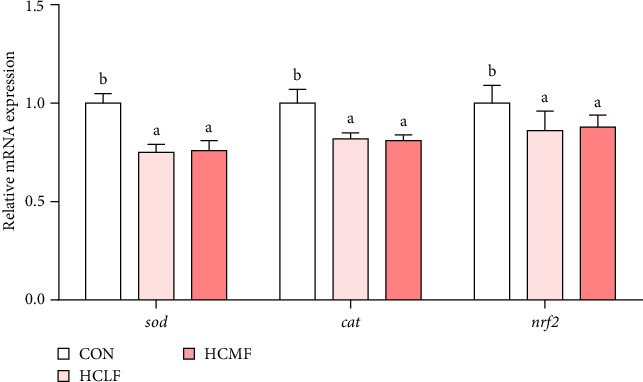
Muscle antioxidant capacity related mRNA expression levels of Nile tilapia. The mRNA values are means (*n* = 3), with their standard deviations (SDs) represented by vertical bars. Bar graphs of the same color, but with different letters are significantly different (*p* < 0.05). *cat*, catalase; *nrf2*, nuclear factor erythroid 2 like 2; *sod*, superoxide dismutase 1.

Both HSLF and HSMF diets significantly reduced the T-AOC, activity of CAT, and activity of SOD (*p* < 0.05; Table 8). The mRNA expression of *sod*, *cat*, and *nrf2* were also downregulated (*p* < 0.05; Figure 5). Compared to the HSLF group, the T-AOC and the activity of SOD was significantly higher when fish fed with HSMF diet (*p* < 0.05; Table 8).

## 4. Discussion

### 4.1. Growth Performance

Lipids are indispensable nutrients for growth and metabolic processes. The rise in the price of feed oil has resulted in a deficiency of lipids in fish feed. In the present study, we used a HSLF diet containing 554 g/kg starch and 28 g/kg fat to simulate the starch and lipid level of commercial feed used in practical production. Results showed that HSLF diet delayed the weight gain of adult tilapia during natural development. A deficiency in dietary fat may result in a deficiency of EFAs, which can affect the absorption and metabolism of other nutrients, potentially leading to the growth retardation [35]. After feeding HSMF diet, the WGR of tilapia reached a level comparable to that observed the CON group. The CF of fish were also increased which may be resulted by the increase of HSI. Under the condition of a certain dietary protein level, the moderate fat supplementation may facilitate a synergistic effect in the utilization of starch and lipids, thus, improving growth performance [36].

Besides, the HSLF diet significantly enhanced the crude fat in muscle. Fish has limited ability to utilize starch. The excessive starch will be converted into fat and transported to other tissues for storage and energy providing when the dietary fat level was insufficient [24]. Studies have found that the muscle glycogen content of tilapia increases with the increase of dietary starch content [37]. However, muscle glycogen content was reduced in tilapia of HSLF group. Tilapia tend to preferentially use lipids to provide energy compared to starch [38]. The lack of dietary fat may have caused glycogenolysis to provide energy and thus contributed to the decline in muscle glycogen content [35].

### 4.2. Flesh Quality of Tilapia

Flesh quality directly affect the preference and acceptance of consumers for aquatic products [39]. In the present study, the flesh quality of tilapia was evaluated from perspectives of texture, nutritional value, flavor, and antioxidant capacity.

#### 4.2.1. Muscle Texture

In the present study, high starch diets caused a decline in hardness, chewiness, springiness, gumminess, and resilience, which was consistent with study on *M. amblycephala* [40]. Since muscle texture is closely related to myofiber [41, 42], muscle histology was further investigated. Results showed high starch diets decreased the myofiber density and sarcomere length which was positive correlated with the hardness and chewiness of muscle [43]. Furthermore, the expression of MRFs family gene were detected. The *myod*, *myog*, *myf5*, and *myf6* play important role in myofiber differentiation, myofiber proliferation, and myogenesis and are involved in the regulation of muscle texture [41]. Similar to previous reports in rainbow trout (*Oncorhynchus mykiss*) and grass carp (*Ctenopharyngodon idellus*), the high starch diets significantly downregulated the expression level of these genes and inhibit the formation of myofiber and myocyte [44, 45]. Moderate fat supplementation also showed improvement in this regard with increased myofiber density and mRNA expression of *myf5* compared to the HSLF diet. Previous studies have shown that the expression levels of MRFs were positive correlated with contents of methionine, cystine, and lysine [46, 47]. HSLF diet, instead of HSMF diet, caused a decrease in amino acids level mentioned above, which may be the reason for the inhibition of MRFs gene expression.

As the main component of connective tissue, collagen protein directly affects muscle hardness. The content of collagen protein was usually expressed by Hyp content [42]. In the present study, both HSLF and HSMF diets decreased the contents of total Hyp, alkaline-soluble Hyp and alkaline-insoluble Hyp. Previous studies on mouse have demonstrated that high carbohydrate could inhibit the type 1 and type 2 procollagen protein of desmocyte, downregulate mRNA expression of *timp2*, and upregulate mRNA expression of *mmp2* [48]. High starch diets might reduce collagen protein synthesis in the way described above. Moreover, HSMF had positive effect on improving the content of total Hyp and alkaline-soluble Hyp compared to HSLF diet. Similar results can be found in study on grass carp that indicated moderate fat level can promote the transcription and translation of collagen protein [45]. The above results suggested excessive starch diets influenced the muscle texture of adult tilapia and moderate fat supplementation promoted the development of myofiber and collagen synthesis.

#### 4.2.2. Nutritional Value

Nutritional value of muscle is mainly determined by the fatty acids and amino acids profiles [49, 50]. Fatty acid composition of muscle is influenced by dietary fat level [51]. Studies have been conducted in a variety of fish, but the results vary due to species and developmental stages [52–54]. In the present study, HSLF diet significantly reduced the contents of *n*−3 PUFAs, especially the contents of EPA and DHA, representing a decline in food quality and unhealthy fatty acid distribution [55, 56]. The biosynthesis of PUFAs in fish, including DHA and EPA, is related to the elongation and desaturation of fatty acids mediated by fatty acid elongase (ELO) and fatty acid desaturation (FAD) enzyme. The activities of ELO and FAD enzymes vary with the potential variation of fatty acid composition caused by increased or reductive dietary fat [57]. Therefore, the significant reduction of PUFAs in the HSLF group may also be due to the inadequate fish oil ingestion which caused deficiency in some fatty acids. Moreover, the contents of SFAs and MUFAs were increased in HSLF group. The high contents of SFAs and MUFAs may lead to fat accumulation and affect muscle texture and taste [17]. The ratio of PUFAs to SFAs and *n*−3 PUFAs to *n*−6 PUFAs, indicators reflecting the nutritional value of dietary foods were significantly lower when fish fed with HSLF diet [58, 59]. Normal PUFAs/SFAs ranges from 0.51−0.56, and ratios of *n*−6/*n*−3 ranges from 0.25−1.00 [60]. Apparently, the original fatty acid balance was disturbed by the HSLF diet due to the lack of fish oil. When tilapia feeding the HSMF diet, the above indices were closer to those of the control group.

The higher proportion of bound EAAs to total bound amino acids in muscle means the higher nutritional value [26]. In the present study, high starch diets did not affect the level of individual amino acid, while total bound EAA were significantly lower when fish feeding the HSLF diet. Meanwhile, tilapia feeding the HSMF diet showed no significant effect on bound EAA compared to the CON group. These results indicated that the moderate fat supplementation is beneficial to the construction of healthier bound amino acid profile, which improved the edibleness of flesh.

#### 4.2.3. Flavor

Flavor of food is produced by the cumulative effect of existing substances [61]. Flesh flavor was explored in terms of fatty acids and amino acids in this study. The proportion for unsaturated fatty acids strongly affect flavor formation, especially fatty acids with more than two double bonds [62]. In the present study, high starch diets reduced levels of PUFAs and *n*−3 PUFAs, while enhanced the level of myristic acid (C14:0), another flavor fatty acid used as flavoring agent in food items [63]. These results suggested that muscle of tilapia feeding high starch diets might form unique flavor unlike CON group. Such difference also existed between HSLF group and HSMF group because of differences in fatty acids levels. Similar results were found in studies on yellow catfish (*Pelteobagrus fulvidraco*) and swimming crab (*Portunus trituberculatus*) [64, 65].

Free amino acids are also important factors of seafood flavor [66]. In the present study, total free EAAs were significantly lower when fish feeding the HSLF diet, while HSMF diet increased the level compared to the CON group, which was consistent with the results of bound amino acids. In addition, the levels of umami amino acids, primarily glutamic acid, as well as sweet amino acids, primarily alanine, lysine, and proline, were significantly decreased when fish fed with the HSLF diet, while the bitter associated amino acids, primarily arginine and histidine were increased [67–69]. After appropriate fat addition, the umami amino acids and sweetish acids returned to normal level. However, there was no such change in bitter amino acids, which indicated that HSMF diet played role in enhancing umami and sweetish taste while also increasing the bitterness of fish muscle. The above results suggested that high starch diets influenced the muscle flavor and nutritional value, and moderate fat supplementation contributed to the formation of healthy fatty acids and amino acids profile.

#### 4.2.4. Antioxidant Capacity

Excessive carbohydrate intake can cause oxidative stress and reduced antioxidant capacity in fish [70, 71]. Fish muscle tissue contains high level of PUFAs, which is one of the reasons that muscle is prone to lipid peroxidation, which cannot only lead to the production of bad odors, but also produce harmful substances that cause protein damage [72]. Oxidative stress can also restrain the synthesis of new collagen mediated by myofibroblasts [73]. Therefore, the decline in antioxidant capacity seriously threatens the flesh quality. In the present study, fish fed with high starch diets also showed significantly lower T-AOC, CAT activity, SOD activity, and expression level of *nrf2*, which was similar to the results of studies on Siberian sturgeon (*Acipenser baerii*) and *M. amblycephala* [74, 75]. HSMF diet increased the T-AOC and SOD activity compared to the HSLF diet. The reason may attribute to inappropriate starch caused abnormal lipid deposition in muscle tissue and enhanced the susceptibility to oxidative stress [76]. Above all, HSLF diets reduced the muscle antioxidant capacity, and moderate fat supplementation could partly alleviate the antioxidant decline.

## 5. Conclusion

HSLF diet could reduce flesh quality by affecting the muscle texture, downregulating the contents of PUFAs, EAAs, and FAAs, and decreasing antioxidant capacity. Moderate fat supplementation could partly alleviate the negative effects caused by HSLF diet specifically by improving the sensory quality and nutritional value of adult tilapia muscle. Further studies are needed to elucidate the behind mechanism and substances that play key role in the regulation of muscle development and nutrients deposition.

## Figures and Tables

**Table 1:** Formulation and proximate analysis of the experimental diets (%).

Ingredient (g/kg)	Diets		
	CON	HSLF	HSMF
Casein	260.00	260.00	260.00
Gelatin	65.00	65.00	65.00
Fish oil	40.00	0.00	40.00
Soybean oil	40.00	30.00	40.00
Corn starch	350.00	550.00	550.00
Micro-cellulose	150.00	50.00	0.00
Bentonite	50.00	0.00	0.00
Vitamin premix^a^	10.00	10.00	10.00
Mineral premix^b^	10.00	10.00	10.00
Ca(H_2_PO_4_)_2_	20.00	20.00	20.00
Choline chloride	2.50	2.50	2.50
Vitamin C	2.50	2.50	2.50
Analyzed nutrients composition (dry matter basis)			
Crude protein (g/kg)	323.07	318.07	317.46
Crude lipid (g/kg)	78.61	28.42	78.17
Ash (g/kg)	78.31	28.15	28.09
Starch (g/kg)	360.47	554.11	563.49
Gross energy (MJ/kg)	19.86	20.42	19.42

Abbreviations: HSLF, high-starch-low-fat; HSMF, high-starch-moderate-fat.

^a^Composition of vitamin premix: vitamin A, 0.8 g; vitamin D_3_, 0.08 g; vitamin E, 20 g; vitamin K_3_, 18.3 g; vitamin B_1_, 10 g; vitamin B_2_, 12.5 g; vitamin B_6_, 8 g; nicotinic acid, 12 g; biotin, 3 g; calcium pantothenate, 20 g; folic acid, 3.2 g; inositol, 406 g. All ingredients were diluted with micro-cellulose to 1 kg.

^b^Composition of mineral premix: C_6_H_10_CaO_6_ 50 g, FeSO_4_ 20 g, MgSO_4_ 100 g, NaH_2_PO_4_ 100 g, NaCl 20 g, AlCl_3_ 0.6 g, KIO_3_ 0.6 g, KCl 40 g, CuSO_4_ 2 g, MnSO_4_ 4 g, ZnSO_4_ 20 g, CoCl_2_ 2 g. All ingredients were diluted with micro-cellulose to 1 kg.

**Table 2:** Real-time quantitative PCR primers used in this study.

Primers	Accession number	Primer sequence		Product length (bp)	*T*_m_ (°C)	Correlation efficiency (%)
*β-Actin*	XM_003443127.5	Forward	TCGTGCGTGACATCAAGGAGAAG	180	60	99.7
		Reverse	CAAGGAAGGAAGGCTGGAAGAGG			

*myod*	NM_001279720.1	Forward	CCTCCTCTTCATCCTCATCCTCC	181	60	96.5
		Reverse	GCGTTGGTCGTCTTCCTCTTG			

*myog*	NM_001279526.1	Forward	GAGGAGCACGCTGATGAACC	185	59	98.5
		Reverse	CGCTTGACGACGACACTCTG			

*myf5*	XM_005456634.3	Forward	GGCGGCTGAAGAAGGTGAAC	121	58	97.8
		Reverse	AGGCTCTCGATGTACTGGATGG			

*myf6*	NM_001282891.1	Forward	CCTCCGCTGACCATTCCACTT	116	60	98.6
		Reverse	GCTGTCGTTGGTGATGCTGTC			

*nrf2*	XM_003447296.5	Forward	GCTGGACTCGCTGAAGGAAGA	184	58	99.2
		Reverse	GCCATCCGTTGACTGCTGAAG			

*cat*	XM_003447521.5	Forward	CCCATCCTTCGTTCATTCCCAGAA	175	58	97.8
		Reverse	GGTGTGAGAGCCGTAGCCATTC			

*sod*	XM_003446807.4	Forward	GCCCACACTTCAATCCCTACAA	219	56	96.7
		Reverse	GGCTCTCTTCATTTCCTCCTTT			

Abbreviations: *cat*, catalase; *myf5*, myogenic factor 5; *myf6*, myogenic factor 6; *myod*, myogenic differentiation 1; *myog*, myogenin; *nrf2*, nuclear factor erythroid 2 like 2; *sod*, superoxide dismutase 1.

**Table 3:** Growth performance of Nile tilapia^1^.

Parameters^2^	Diets		
	CON	HSLF	HSMF
IBW (g)^3^	168.63 ± 2.86	168.63 ± 2.75	168.47 ± 1.55
FBW (g)^3^	502.03 ± 27.68^b^	396.55 ± 9.68^a^	497.19 ± 17.71^b^
WGR (%)^3^	197.62 ± 12.97^b^	135.19 ± 6.69^a^	195.17 ± 12.03^b^
FCR^3^	1.12 ± 0.04^a^	1.55 ± 0.04^b^	1.16 ± 0.08^a^
HSI (%)^3^	1.82 ± 0.03^a^	1.96 ± 0.09^ab^	2.02 ± 0.04^b^
MFI (%)^3^	2.14 ± 0.18^a^	2.78 ± 0.07^b^	2.52 ± 0.11^b^
VSI (%)^3^	8.05 ± 0.60^a^	8.90 ± 0.58^b^	9.25 ± 0.80^b^
CF (g/cm^3^)	3.89 ± 0.07^a^	3.78 ± 0.01^a^	4.10 ± 0.03^b^

Abbreviations: HSLF, high-starch-low-fat; HSMF, high-starch-moderate-fat; SD, standard deviation.

^1^Data are presented as mean ± SD (*n* = 3). Different superscript letters (a and b) within the same row indicate significant differences (*p* < 0.05). Each row sharing the same superscript letter or absence of superscript are not significantly different (*p* > 0.05).

^2^IBW, initial body weight; FBW, final body weight; WGR, weight gain rate; CF, condition factor; FCR, feed conversion ratio; HSI, Hepatosomatic index, MFI, mesenteric fat index; VSI, viscerosomatic index.

^3^Data are originated from our preliminary findings [34] (https://doi.org/10.1016/j.aqrep.2025.102639).

**Table 4:** Muscle proximate composition of Nile tilapia^1^.

Parameters (g/kg)	Diets		
	CON	HSLF	HSMF
Crude protein	177.03 ± 5.34	179.97 ± 3.45	176.15 ± 3.69
Crude fat	24.67 ± 2.83^a^	32.87 ± 3.93^b^	24.03 ± 2.59^a^
Moisture	769.89 ± 6.45	762.00 ± 8.31	769.4 ± 7.68
Ash	12.18 ± 0.70	12.37 ± 0.87	12.32 ± 0.57
Glycogen	0.99 ± 0.14^b^	0.66 ± 0.09^a^	1.46 ± 0.24^c^

Abbreviations: HSLF, high-starch-low-fat; HSMF, high-starch-moderate-fat; SD, standard deviation.

^1^Data are presented as mean ± SD (*n* = 3). Different superscript letters (a, b, and c) within the same row indicate significant differences (*p* < 0.05). Each row sharing the same superscript letter or absence of superscript are not significantly different (*p* > 0.05).

**Table 5:** Muscle texture of Nile tilapia^1^.

Parameters	Diets		
	CON	HSLF	HSMF
Hardness (gf)	2361.70 ± 113.44^b^	2147.60 ± 89.42^a^	2157.20 ± 108.03^a^
Chewiness (gf)	403.88 ± 10.45^b^	372.12 ± 7.86^a^	370.76 ± 9.87^a^
Springiness	0.55 ± 0.03^b^	0.48 ± 0.04^a^	0.51 ± 0.04^a^
Gumminess	721.96 ± 43.16^b^	650.70 ± 29.02^a^	635.05 ± 24.43^a^
Resilience	0.36 ± 0.05^b^	0.29 ± 0.03^a^	0.33 ± 0.06^b^
Water holding capacity (%)	87.21 ± 1.18	86.48 ± 0.75	86.80 ± 0.81

Abbreviations: HSLF, high-starch-low-fat; HSMF, high-starch-moderate-fat; SD, standard deviation.

^1^Data are presented as mean ± SD (*n* = 3). Different superscript letters (a and b) within the same row indicate significant differences (*p* < 0.05). Each row sharing the same superscript letter or absence of superscript are not significantly different (*p* > 0.05).

**Table 6:** Muscle fatty acid composition of Nile tilapia^1^.

Parameters (mg/kg)	Diets		
	CON	HSLF	HSMF
C12:0	0.031 ± 0.00	0.035 ± 0.01	0.036 ± 0.01
C14:0	0.62 ± 0.03^a^	1.07 ± 0.04^c^	0.84 ± 0.04^b^
C15:0	0.103 ± 0.00^b^	0.06 ± 0.01^a^	0.089 ± 0.01^b^
C16:0	6.76 ± 0.51^a^	9.73 ± 1.08^b^	5.93 ± 0.58^a^
C17:0	0.105 ± 0.0^c^	0.074 ± 0.00^a^	0.098 ± 0.00^b^
C18:0	1.94 ± 0.06^b^	2.89 ± 0.04^c^	1.72 ± 0.03^a^
C20:0	0.093 ± 0.00^b^	0.095 ± 0.00^b^	0.082 ± 0.01^a^
C22:0	0.064 ± 0.01	0.055 ± 0.01	0.055 ± 0.00
SFAs	9.68 ± 0.53^a^	14.01 ± 1.59^b^	8.85 ± 0.63^a^
C14:1*n*−5	0.046 ± 0.00	0.044 ± 0.01	0.041 ± 0.01
C16:1*n*−7	1.38 ± 0.18^a,b^	1.77 ± 0.22^b^	1.23 ± 0.16^a^
C17:1*n*−7	0.054 ± 0.01	0.056 ± 0.01	0.054 ± 0.00
C18:1*n*−9	7.94 ± 0.89^a^	12.12 ± 1.66^b^	6.96 ± 0.50^a^
C20:1*n*−9	0.42 ± 0.04^a^	0.63 ± 0.08^b^	0.36 ± 0.02^a^
C22:1*n*−9	0.102 ± 0.01^b^	0.111 ± 0.00^b^	0.072 ± 0.01^a^
C24:1*n*−9	0.084 ± 0.01^b^	0.062 ± 0.01^a^	0.064 ± 0.01^a^
MUFAs	10.02 ± 1.11^a^	14.79 ± 1.99^b^	8.78 ± 0.65^a^
C18:2*n*−6	4.10 ± 0.39	3.77 ± 0.25	3.45 ± 0.23
C18:3*n*−6	0.16 ± 0.00^b^	0.25 ± 0.01^c^	0.13 ± 0.00^a^
C20:2*n*−6	0.20 ± 0.02	0.19 ± 0.01	0.18 ± 0.01
C20:3*n*−6	0.23 ± 0.03^a,b^	0.30 ± 0.01^b^	0.21 ± 0.04^a^
C20:4*n*−6	0.52 ± 0.01^b^	0.69 ± 0.04^c^	0.45 ± 0.00^a^
*n*−6 PUFAs	5.21 ± 0.26	5.19 ± 0.16	4.42 ± 0.12
C18:3*n*−3	0.39 ± 0.04^b^	0.30 ± 0.03^a^	0.32 ± 0.01^a,b^
C20:3*n*−3	0.083 ± 0.01	0.079 ± 0.02	0.071 ± 0.01
C20:5*n*−3	0.17 ± 0.01^b^	0.13 ± 0.01^a^	0.14 ± 0.01^a,b^
C22:6*n*−3	2.07 ± 0.10^c^	0.58 ± 0.04^a^	1.81 ± 0.03^b^
*n*−3 PUFAs	2.71 ± 0.05^c^	1.09 ± 0.06^a^	2.34 ± 0.01^b^
PUFAs	7.92 ± 0.44^b^	6.28 ± 0.38^a^	6.77 ± 0.19^a^
*n*−3 PUFAs/*n*−6 PUFAs	0.52 ± 0.03^b^	0.21 ± 0.01^a^	0.53 ± 0.01^b^
PUFAs/SFAs	0.82 ± 0.03^b^	0.45 ± 0.01^a^	0.77 ± 0.04^b^

Abbreviations: HSLF, high-starch-low-fat; HSMF, high-starch-moderate-fat; MUFA, monounsaturated fatty acid; PUFA, polyunsaturated fatty acid; SD, standard deviation; SFA, saturated fatty acid.

^1^Data are presented as mean ± SD (*n* = 3). Different superscript letters (a, b, and c) within the same row indicate significant differences (*p*  < 0.05). Each row sharing the same superscript letter or absence of superscript are not significantly different (*p* > 0.05).

**Table 7:** Muscle free amino acid composition of Nile tilapia^1^.

Parameters (mg/kg)	Diets		
	CON	HSLF	HSMF
EAAs			
Arginine	12.07 ± 1.47^a^	16.49 ± 0.82^b^	16.59 ± 0.95^b^
Histidine	211.00 ± 2.81^a^	247.04 ± 9.72^c^	228.58 ± 9.66^b^
Isoleucine	25.73 ± 0.77^c^	14.48 ± 0.36^a^	20.40 ± 1.08^b^
Leucine	25.88 ± 1.04^a^	25.41 ± 1.11^a^	33.78 ± 0.54^b^
Lysine	122.34 ± 3.36^b^	79.39 ± 3.35^a^	147.80 ± 3.20^c^
Methionine	19.75 ± 0.59^b^	14.57 ± 1.08^a^	21.03 ± 1.07^b^
Phenylalanine	20.30 ± 1.58	18.82 ± 0.77	18.69 ± 1.14
Threonine	144.60 ± 7.07^b^	122.26 ± 6.11^a^	120.66 ± 6.57^a^
Valine	34.23 ± 1.46^b^	29.12 ± 1.49^a^	35.80 ± 1.10^b^
NEAA			
Alanine	287.79 ± 5.06^b^	89.69 ± 3.69^a^	304.02 ± 6.31^c^
Aspartate	75.56 ± 5.33^a^	73.81 ± 5.11^a^	86.06 ± 4.53^b^
Glycine	396.73 ± 11.26^a^	502.43 ± 8.69^c^	431.07 ± 19.77^b^
Glutamate	169.50 ± 4.80^c^	92.18 ± 3.40^a^	128.06 ± 6.22^b^
Proline	1292.18 ± 34.88^b^	789.61 ± 72.91^a^	1242.58 ± 46.76^b^
Serine	143.83 ± 8.14^b^	290.21 ± 12.72^c^	118.15 ± 7.94^a^
Tyrosine	28.27 ± 0.94^c^	21.96 ± 1.12^b^	18.59 ± 0.91^a^

Abbreviations: EAA, essential amino acid; HSLF, high-starch-low-fat; HSMF, high-starch-moderate-fat; NEAA, non-EAA; SD, standard deviation.

^1^Data are presented as mean ± SD (*n* = 3). Different superscript letters (a, b, and c) within the same row indicate significant differences (*p* < 0.05). Each row sharing the same superscript letter or absence of superscript are not significantly different (*p*  > 0.05).

**Table 8:** Muscle antioxidant capacity of Nile tilapia^1^.

Parameters^2^	Diets		
	CON	HSLF	HSMF
T-AOC (mmol/mgprot)	0.31 ± 0.031^c^	0.19 ± 0.03^a^	0.26 ± 0.03^b^
CAT (U/mgprot)	12.63 ± 1.55^c^	8.56 ± 1.01^b^	6.23 ± 0.99^a^
SOD (U/mgprot)	19.18 ± 0.51^c^	16.61 ± 0.39^a^	18.3 ± 0.44^b^

Abbreviations: HSLF, high-starch-low-fat; HSMF, high-starch-moderate-fat; SD, standard deviation.

^1^Data are presented as mean ± SD (*n* = 3). Different superscript letters (a, b, and c) within the same row indicate significant differences (*p* < 0.05). Each row sharing the same superscript letter or absence of superscript are not significantly different (*p* > 0.05).

^2^T-AOC, total antioxidant capacity; CAT, catalase; SOD, superoxide dismutase.

## Data Availability

The data that support the findings of this study are available on request from the corresponding author. The data are not publicly available due to privacy or ethical restrictions.

## Supporting Information

Supporting Information Supporting Infomation Figure S1. Muscle bound amino acid levels of Nile tilapia. (A) Total amino acids, (B) essential amino acids, and (C) nonessential amino acids. Data values are means (*n* = 3), with their standard deviations represented by vertical bars. Bar graphs of the same color but with different letters are significantly different (*p* < 0.05). Supporting Information Table S1. Muscle bound amino acid composition of Nile tilapia.

## References

[1] Borges N., Keller-Costa T., Sanches-Fernandes G. M. M., Louvado Aónio, Gomes N. C. M., Costa R. (2021). Bacteriome Structure, Function, and Probiotics in Fish Larviculture: The Good, the Bad, and the Gaps. *Annual Review of Animal Biosciences*.

[2] Ng W.-K., Romano N. (2013). A Review of the Nutrition and Feeding Management of Farmed Tilapia Throughout the Culture Cycle. *Reviews In Aquaculture*.

[3] He C., Cao J., Bao Y., Sun Z., Liu Z., Li C. (2021). Characterization of Lipid Profiling in Three Parts (Muscle, Head and Viscera) of Tilapia (*Oreochromis niloticus*) Using Lipidomics With UPLC-ESI-Q-TOF-MS. *Food Chemistry*.

[4] Zheng J., Zhang W., Dan Z. (2023). Effects of Fish Meal Replaced by Methanotroph Bacteria Meal (*Methylococcus capsulatus*) on Growth, Body Composition, Antioxidant Capacity, Amino Acids Transporters and Protein Metabolism of Turbot Juveniles (*Scophthalmus maximus* L.). *Aquaculture*.

[5] Boonanuntanasarn S., Jangprai A., Kumkhong S. (2018). Adaptation of Nile Tilapia (*Oreochromis niloticus*) to Different Levels of Dietary Carbohydrates: New Insights From a Long Term Nutritional Study. *Aquaculture*.

[6] Gominho-Rosa M. d C., Rodrigues A. P. O., Mattioni B., de Francisco A., Moraes G., Fracalossi D. M. (2015). Comparison Between the Omnivorous Jundiá Catfish (*Rhamdia quelen*) and Nile Tilapia (*Oreochromis niloticus*) on the Utilization of Dietary Starch Sources: Digestibility, Enzyme Activity and Starch Microstructure. *Aquaculture*.

[7] Liu W., Lu X., Jiang M. (2020). Effects of Dietary Niacin on Liver Health in Genetically Improved Farmed Tilapia (*Oreochromis niloticus*). *Aquaculture Reports*.

[8] Zhao W., Xie J.-J., Fang H.-H., Liu Y.-J., Tian L.-X., Niu J. (2020). Effects of Corn Starch Level on Growth Performance, Antioxidant Capacity, Gut Morphology and Intestinal Microflora of Juvenile Golden Pompano, *Trachinotus ovatus*. *Aquaculture*.

[9] Jeong S.-M., Khosravi S., Lee S. Y., Kim K.-W., Lee B.-J., Lee S.-M. (2021). Evaluation of the Three Different Sources of Dietary Starch in an Extruded Feed for Juvenile Olive Flounder, *Paralichthys olivaceus*. *Aquaculture*.

[10] Li Y., Lu X., Gao W. (2022). The Effect of Dietary Paeonol on Growth Performance, Antioxidant Enzyme Activities and Gene Expressions of Genetic Improvement of Farmed Tilapia Juveniles (*Oreochromis niloticus*). *Aquaculture Reports*.

[11] Yu L., Tian J., Zhang C. (2022). Acetylferulic Paeonol Ester: A New Feed Additive Reduces Lipid Accumulation in the Liver of Nile Tilapia (*Oreochromis niloticus*) by Modulating Lipid and Glucose Metabolism. *Aquaculture*.

[12] Huang Y., Wang M., Pan J. (2024). Dietary Glutamine Supplementation Improves the Osmoregulatory Capacity and Reduces Oxidative Stress Induced by Hyperosmotic Stress in Nile Tilapia (*Oreochromis niloticus*). *Aquaculture Reports*.

[13] Zhou Y.-L., He G.-L., Jin T. (2021). High Dietary Starch Impairs Intestinal Health and Microbiota of Largemouth Bass, *Micropterus salmoides*. *Aquaculture*.

[14] Gou N., Chang Z., Deng W., Ji H., Zhou J. (2019). Effects of Dietary Lipid Levels on Growth, Fatty Acid Composition, Antioxidant Status and Lipid Metabolism in Juvenile *Onychostoma macrolepis*. *Aquaculture Research*.

[15] Zhan Q., Han T., Li X. (2020). Effects of Dietary Carbohydrate Levels on Growth, Body Composition, and Gene Expression of Key Enzymes Involved in Hepatopancreas Metabolism in Mud Crab *Scylla paramamosain*. *Aquaculture*.

[16] Zhang X., Jin M., Luo J. (2022). Effects of Dietary Carbohydrate Levels on the Growth and Glucose Metabolism of Juvenile Swimming Crab, (*Portunus trituberculatus*). *Aquaculture Nutrition*.

[17] Wang B.-K., Liu W.-B., Xu C. (2017). Dietary Carbohydrate Levels and Lipid Sources Modulate the Growth Performance, Fatty Acid Profiles and Intermediary Metabolism of Blunt Snout Bream *Megalobrama amblycephala* in an Interactive Pattern. *Aquaculture*.

[18] Li J. N., Xu Q. Y., Wang C. A., Wang L. S., Zhao Z. G., Luo L. (2016). Effects of Dietary Glucose and Starch Levels on the Growth, Haematological Indices and Hepatic Hexokinase and Glucokinase mRNA Expression of Juvenile Mirror Carp (*Cyprinus carpio*). *Aquaculture Nutrition*.

[19] Li S., Yang Z., Tian H., Ren S., Zhang W., Wang A. (2022). Effects of Dietary Carbohydrate-to-Lipid Ratios on Growth Performance, Intestinal Digestion, Lipid and Carbohydrate Metabolism of Red Swamp Crayfish (*Procambarus clarkii*). *Aquaculture Reports*.

[20] Regost C., Arzel J., Cardinal M., Robin J., Laroche M., Kaushik S. J. (2001). Dietary Lipid Level, Hepatic Lipogenesis and Flesh Quality in Turbot (*Psetta maxima*). *Aquaculture*.

[21] Turchini G. M., Torstensen B. E., Ng W.-K. (2009). Fish Oil Replacement in Finfish Nutrition. *Reviews In Aquaculture*.

[22] Kiron V. (2012). Fish Immune System and Its Nutritional Modulation for Preventive Health Care. *Animal Feed Science and Technology*.

[23] Zuo R., Ai Q., Mai K. (2012). Effects of Dietary n-3 Highly Unsaturated Fatty Acids on Growth, Nonspecific Immunity, Expression of Some Immune Related Genes and Disease Resistance of Large Yellow Croaker (*Larmichthys crocea*) Following Natural Infestation of Parasites (*Cryptocaryon irritans*). *Fish & Shellfish Immunology*.

[24] Xie D., Yang L., Yu R. (2017). Effects of Dietary Carbohydrate and Lipid Levels on Growth and Hepatic Lipid Deposition of Juvenile Tilapia, *Oreochromis niloticus*. *Aquaculture*.

[25] Kabir K. A., Verdegem M. C. J., Verreth J. A. J., Phillips M. J., Schrama J. W. (2020). Effect of Dietary Carbohydrate to Lipid Ratio on Performance of Nile Tilapia and Enhancement of Natural Food in Pond Aquaculture. *Aquaculture Research*.

[26] Wu H.-X., Li W.-J., Shan C.-J. (2021). Oligosaccharides Improve the Flesh Quality and Nutrition Value of Nile Tilapia Fed With High Carbohydrate Diet. *Food Chemistry: Molecular Sciences 3*.

[27] Tian J., Wu F., Yang C.-G., Jiang M., Liu W., Wen H. (2015). Dietary Lipid Levels Impact Lipoprotein Lipase, Hormone-Sensitive Lipase, and Fatty Acid Synthetase Gene Expression in Three Tissues of Adult GIFT Strain of Nile Tilapia, *Oreochromis niloticus*. *Fish Physiology and Biochemistry*.

[28] Jiang M., Liu W., Lu X. (2018). Dietary Phosphatidylcholine Impacts on Growth Performance and Lipid Metabolism in Adult Genetically Improved Farmed Tilapia (GIFT) Strain of Nile Tilapia *Oreochromis niloticus*. *British Journal of Nutrition*.

[29] Lin J., Liao Y., Li X. (2023). Effects of Dietary Creatine Levels on the Growth, Muscle Energy Metabolism and Meat Quality of Spotted Seabass (*Lateolabrax maculatus*) Fed Low-Fishmeal Diets. *Aquaculture*.

[30] Zhang K., Ai Q., Mai K. (2013). Effects of Dietary Hydroxyproline on Growth Performance, Body Composition, Hydroxyproline and Collagen Concentrations in Tissues in Relation to Prolyl 4-Hydroxylase *α*(I) Gene Expression of Juvenile Turbot, *Scophthalmus maximus* L. Fed High Plant Protein Diets. *Aquaculture*.

[31] Zhang J., Wang Y., Liu J. (2023). Effects of Fecal Bacteria on Growth, Digestive Capacity, Antioxidant Capacity, Intestinal Health of Large Yellow Croaker (*Larimichthys crocea*) Larvae. *Aquaculture*.

[32] Cao M., Xie N., Zhang J. (2024). Dietary Supplementation With Succinic Acid Improves Growth Performance and Flesh Quality of Adult Nile Tilapia (*Oreochromis niloticus*) Fed a High-Carbohydrate Diet. *Animal Nutrition*.

[33] Livak K. J., Schmittgen T. D. (2001). Analysis of Relative Gene Expression Data Using Real-Time Quantitative PCR and the 2^−ΔΔC^_T_ Method. *Methods*.

[34] Xie N., Zhang J., Jiang M. (2025). Effects of Lipid Levels on Growth Performance and Glucose and Lipid Metabolism of Adult Nile Tilapia (*Oreochromis niloticus*) Fed With High Carbohydrate Diets. *Aquaculture Reports*.

[35] Liu H., Yang J. J., Dong X. H. (2020). Effects of Different Dietary Carbohydrate-to-Lipid Ratios on Growth, Plasma Biochemical Indexes, Digestive, and Immune Enzymes Activities of Sub-Adult Orange-Spotted Grouper *Epinephelus coioides*. *Fish Physiology and Biochemistry*.

[36] Bell J. G., Tocher D. R., Farndale B. M., Cox D. I., McKinney R. W., Sargent J. R. (1997). The Effect of Dietary Lipid on Polyunsaturated Fatty Acid Metabolism in Atlantic Salmon (*Salmo salar*) Undergoing Parr-Smolt Transformation. *Lipids*.

[37] Sargent J., Bell G., McEvoy L., Tocher D., Estevez A. (1999). Recent Developments in the Essential Fatty Acid Nutrition of Fish. *Aquaculture*.

[38] Li X., Han T., Zheng S., Wu G. (2022). Hepatic Glucose Metabolism and Its Disorders in Fish. *Recent Advances in Animal Nutrition and Metabolism*.

[39] Huang T., Guo B., Zheng J. (2024). Combined Supplementation of Hydroxyproline and Vitamin C Improved the Growth and Flesh Quality of Pacific White Shrimp (*Litopenaeus vanname*) Cultured in Low Salinity Water. *Aquaculture and Fisheries*.

[40] Bao S.-T., Liu X.-C., Huang X.-P. (2022). Magnesium Supplementation in High Carbohydrate Diets: Implications on Growth, Muscle Fiber Development and Flesh Quality of *Megalobrama amblycephala*. *Aquaculture Reports*.

[41] Koganti P., Yao J., Cleveland B. M. (2021). Molecular Mechanisms Regulating Muscle Plasticity in Fish. *Animals*.

[42] Cheng X., Li M., Leng X. (2021). Creatine Improves the Flesh Quality of Pacific White Shrimp (*Litopenaeus vannamei*) Reared in Freshwater. *Food Chemistry*.

[43] Johnston I. A., Manthri S., Bickerdike R. (2004). Growth Performance, Muscle Structure and Flesh Quality in Out-of-Season Atlantic Salmon (*Salmo salar*) Smolts Reared Under Two Different Photoperiod Regimes. *Aquaculture*.

[44] Chapalamadugu K. C., Robison B. D., Drew R. E. (2009). Dietary Carbohydrate Level Affects Transcription Factor Expression that Regulates Skeletal Muscle Myogenesis in Rainbow Trout. *Comparative Biochemistry and Physiology Part B: Biochemistry and Molecular Biology*.

[45] Wu J.-Y., Feng L., Wu P. (2022). Modification of Beneficial Fatty Acid Composition and Physicochemical Qualities in the Muscle of Sub-Adult Grass Carp (*Ctenopharyngodon idella*): The Role of Lipids. *Aquaculture*.

[46] Harthan L. B., McFarland D. C., Velleman S. G. (2014). The Effect of Nutritional Status and Myogenic Satellite Cell Age on Turkey Satellite Cell Proliferation, Differentiation, and Expression of Myogenic Transcriptional Regulatory Factors and Heparan Sulfate Proteoglycans Syndecan-4 and Glypican-11. *Poultry Science*.

[47] Powell D. J., McFarland D. C., Cowieson A. J., Muir W. I., Velleman S. G. (2014). The Effect of Nutritional Status on Myogenic Gene Expression of Satellite Cells Derived From Different Muscle Types. *Poultry Science*.

[48] Quaglino D., Sartor L., Garbisa S. (2005). Dermal Fibroblasts From Pseudoxanthoma Elasticum Patients Have Raised MMP-2 Degradative Potential. *Biochimica et Biophysica Acta (BBA) - Molecular Basis of Disease*.

[49] Jiang W.-D., Wen H.-L., Liu Y. (2016). Enhanced Muscle Nutrient Content and Flesh Quality, Resulting From Tryptophan, Is Associated With Anti-Oxidative Damage Referred to the Nrf2 and TOR Signalling Factors in Young Grass Carp (*Ctenopharyngodon idella*): Avoid Tryptophan Deficiency or Excess. *Food Chemistry*.

[50] Cai L., Tong F., Tang T. (2021). Comparative Evaluation of Nutritional Value and Flavor Quality of Muscle in Triploid and Diploid Common Carp: Application of Genetic Improvement in Fish Quality. *Aquaculture*.

[51] Xu R., Hung S. S. O., Bruce German J. (1993). White Sturgeon Tissue Fatty Acid Compositions Are Affected by Dietary Lipids^1 2^. *The Journal of Nutrition*.

[52] Li Y., Liang X., Zhang Y., Gao J. (2016). Effects of Different Dietary Soybean Oil Levels on Growth, Lipid Deposition, Tissues Fatty Acid Composition and Hepatic Lipid Metabolism Related Gene Expressions in Blunt Snout Bream (*Megalobrama amblycephala*) Juvenile. *Aquaculture*.

[53] Takeuchi T., Watanabe T. (1976). Nutritive Value of *ω*3 Highly Unsaturated Fatty Acids in Pollock Liver Oil for Rainbow Trout. *Nippon Suisan Gakkaishi*.

[54] Radunz-Neto J., Corraze G., Bergot P., Kaushik S. J. (1996). Estimation of Essential Fatty Acid Requirements of Common Carp Larvae Using Semi-Purified Artificial Diets. *Archiv für Tierernaehrung*.

[55] Kris-Etherton P. M., Harris W. S., Appel L. J. (2002). Fish Consumption, Fish Oil, Omega-3 Fatty Acids, and Cardiovascular Disease. *Circulation*.

[56] Calder P. C. (2013). Omega-3 Polyunsaturated Fatty Acids and Inflammatory Processes: Nutrition or Pharmacology?. *British Journal of Clinical Pharmacology*.

[57] Castro L. F. C., Tocher D. R., Monroig O. (2016). Long-Chain Polyunsaturated Fatty Acid Biosynthesis in Chordates: Insights into the Evolution of Fads and Elovl Gene Repertoire. *Progress in Lipid Research*.

[58] Chen J., Liu H. (2020). Nutritional Indices for Assessing Fatty Acids: A Mini-Review. *International Journal of Molecular Sciences*.

[59] Duarte F. O. S., de Paula F. G., Prado C. S. (2021). Better Fatty Acids Profile in Fillets of Nile Tilapia (*Oreochromis niloticus*) Supplemented With Fish Oil. *Aquaculture*.

[60] Tonial I., Oliveira D., Coelho A. (2014). Quantification of Essential Fatty Acids and Assessment of the Nutritional Quality Indexes of Lipids in Tilapia Alevins and Juvenile Tilapia Fish (*Oreochromis niloticus*). *Journal of Food Research*.

[61] Ganguly S., Mahanty A., Mitra T., Mohanty S., Das B. K., Mohanty B. P. (2018). Nutrigenomic Studies on Hilsa to Evaluate Flesh Quality Attributes and Genes Associated With Fatty Acid Metabolism From the Rivers Hooghly and Padma. *Food Research International*.

[62] Wood J. D., Richardson R. I., Nute G. R. (2004). Effects of Fatty Acids on Meat Quality: A Review. *Meat Science*.

[63] Mohanty B. P., Ganguly S., Mahanty A. (2016). DHA and EPA Content and Fatty Acid Profile of 39 Food Fishes From India. *BioMed Research International*.

[64] Sun P., Jin M., Jiao L. (2020). Effects of Dietary Lipid Level on Growth, Fatty Acid Profiles, Antioxidant Capacity and Expression of Genes Involved in Lipid Metabolism in Juvenile Swimming Crab, *Portunus trituberculatus*. *British Journal of Nutrition*.

[65] Fei S., Chen Z., Duan Y. (2024). Growth, Reproduction, Fatty Acid Profiles and Offspring Performance of Broodstock Yellow Catfish *Pelteobagrus fulvidraco* Fed Diets With Different Lipid Levels. *Aquaculture*.

[66] Zang J., Yu D., Zhang P., Xu Y., Xia W. (2022). The Key Enzymes and Flavor Precursors Involved in Formation of Characteristic Flavor Compounds of Low-Salt Fermented Common Carp (*Cyprinus carpio* L.). *LWT*.

[67] Uchida M., Kurushima H., Ishihara K. (2017). Characterization of Fermented Seaweed Sauce Prepared From Nori (*Pyropia yezoensis*). *Journal Of Bioscience and Bioengineering*.

[68] Milinovic J., Mata P., Diniz M., Noronha J. P. (2021). Umami Taste in Edible Seaweeds: The Current Comprehension and Perception. *International Journal of Gastronomy and Food Science*.

[69] Nie J., Fu X., Wang L., Xu J., Gao X. (2023). Impact of Monascus Purpureus Fermentation on Antioxidant Activity, Free Amino Acid Profiles and Flavor Properties of Kelp (*Saccharina japonica*). *Food Chemistry*.

[70] Zhang Q., Chen Y., Xu W., Zhang Y. (2021). Effects of Dietary Carbohydrate Level on Growth Performance, Innate Immunity, Antioxidant Ability and Hypoxia Resistant of Brook Trout *Salvelinus fontinalis*. *Aquaculture Nutrition*.

[71] Han H., Wang Z., Wang J. (2021). Impact of High Dietary Cornstarch Level on Growth, Antioxidant Response, and Immune Status in GIFT Tilapia *Oreochromis niloticus*. *Scientific Reports*.

[72] Jiang W.-D., Feng L., Liu Y. (2010). Lipid Peroxidation, Protein Oxidant and Antioxidant Status of Muscle, Intestine and Hepatopancreas for Juvenile Jian Carp (*Cyprinus carpio* Var. Jian) Fed Graded Levels of Myo-Inositol. *Food Chemistry*.

[73] Archile-Contreras A. C., Purslow P. P. (2011). Oxidative Stress May Affect Meat Quality by Interfering With Collagen Turnover by Muscle Fibroblasts. *Food Research International*.

[74] Li X.-F., Liu W.-B., Lu K.-L., Xu W.-N., Wang Y. (2012). Dietary Carbohydrate/Lipid Ratios Affect Stress, Oxidative Status and Non-Specific Immune Responses of Fingerling Blunt Snout Bream, *Megalobrama amblycephala*. *Fish & Shellfish Immunology*.

[75] Babaei S., Abedian-Kenari A., Hedayati M., Yazdani-Sadati M. A. (2017). Growth Response, Body Composition, Plasma Metabolites, Digestive and Antioxidant Enzymes Activities of Siberian Sturgeon (*Acipenser baerii*, Brandt, 1869) Fed Different Dietary Protein and Carbohydrate: Lipid Ratio. *Aquaculture Research*.

[76] Sabzi E., Mohammadiazarm H., Salati A. P. (2017). Effect of Dietary l-Carnitine and Lipid Levels on Growth Performance, Blood Biochemical Parameters and Antioxidant Status in Juvenile Common Carp (*Cyprinus carpio*). *Aquaculture*.

