# Supplementary material for: Appropriate Fat Supplementation in High-Starch Diets Involved in the Modification of Fatty Acids Profile, Amino Acids Composition, and Antioxidant Capacity of Adult Nile Tilapia (Oreochromis niloticus) Muscle
Source: Aquac Nutr. 2025 Mar 20;2025:7139771. doi: 10.1155/anu/7139771 (PMC11949607; doi:10.1155/anu/7139771)
Supplement: Supporting Information — Supporting Infomation Figure S1. Muscle bound amino acid levels of Nile tilapia. (A) Total amino acids, (B) essential amino acids, and (C) nonessential amino acids. Data values are means (n = 3), with their standard deviations represented by vertical bars. Bar graphs of the same color but with different letters are significantly different (p < 0.05). Supporting Information Table S1. Muscle bound amino acid composition of Nile tilapia. [file 7139771.f1.docx]

Supplementary Table 1 Muscle bound amino acid composition of Nile tilapia ^a^

| Parameters (g/kg) | Diets | | |
| --- | --- | --- | --- |
|  | CON | HSLF | HSMF |
| Essential amino acids |  |  |  |
| Arginine | 11.55±0.49 | 10.61±0.18 | 11.24±00.71 |
| Histidine | 5.05±0.12 | 4.69±0.30 | 4.89±0.42 |
| Isoleucine | 6.91±0.29 | 6.48±0.44 | 6.62±0.64 |
| Leucine | 14.41±0.26 | 14.55±0.72 | 14.38±0.52 |
| Lysine | 16.83±0.56 | 16.84±0.44 | 17.42±0.93 |
| Methionine | 4.42±0.21 | 4.13±0.35 | 4.15±0.38 |
| Phenylalanine | 8.54±0.19 | 7.68±0.10 | 8.32±0.81 |
| Threonine | 9.31±0.28 | 9.73±0.26 | 9.35±0.45 |
| Valine | 9.36±0.25 | 9.26±0.85 | 9.39±0.44 |
| Non-essential amino acid |  |  |  |
| Alanine | 10.57±0.24 | 10.88±0.15 | 10.61±0.25 |
| Aspartate | 16.52±0.53 | 16.95±0.43 | 17.09±0.81 |
| Cysteine | 0.91±0.09 | 0.88±0.05 | 0.93±0.08 |
| Glycine | 10.31±0.46 | 11.01±0.53 | 10.55±0.88 |
| Glutamate | 22.15±0.58 | 22.54±0.13 | 22.62±0.77 |
| Proline | 8.36±0.19 | 8.94±0.66 | 8.77±0.09 |
| Serine | 8.05±0.19 | 8.19±0.09 | 8.13±0.29 |
| Tyrosine | 6.53±0.31 | 5.94±0.69 | 6.10±0.72 |

^a^ Data are presented as mean ± SD (n=3). Each row sharing the same superscript letter or absence of superscript are not significantly different determined by Tukey’s test (*P* > 0.05). SD: standard deviation.





Supplementary Fig. 1. Muscle bound amino acid levels of Nile tilapia. (A) total amino acids, (B) essential amino acids and (C) non-essential amino acids. Data values are means (n = 3), with their standard deviations represented by vertical bars. Bar graphs of the same color but with different letters are significantly different (*P* < 0.05).
